# Voltage-driven translocation behaviors of IgG molecule through nanopore arrays

**DOI:** 10.1186/1556-276X-8-229

**Published:** 2013-05-15

**Authors:** Lei Liu, Bing Wang, Jingjie Sha, Yue Yang, Yaozong Hou, Zhonghua Ni, Yunfei Chen

**Affiliations:** 1 Jiangsu Key Laboratory for Design and Manufacture of Micro-Nano Biomedical Instruments, School of Mechanics, Southeast University, Nanjing 210096, People's Republic of China

**Keywords:** Nanopore arrays, Biosensing, Simulation

## Abstract

Nanopore-based biosensing has attracted more and more interests in the past years, which is also regarded as an emerging field with major impact on bio-analysis and fundamental understanding of nanoscale interactions down to single-molecule level. In this work, the voltage-driven translocation properties of goat antibody to human immunoglobulin G (IgG) are investigated using nanopore arrays in polycarbonate membranes. Obviously, the background ionic currents are modulated by IgG molecules for their physical place-holding effect. However, the detected ionic currents do ‘not’ continuously decrease as conceived; the currents first decrease, then increase, and finally stabilize with increasing IgG concentration. To understand this phenomenon, a simplified model is suggested, and the calculated results contribute to the understanding of the abnormal phenomenon in the actual ionic current changing tendency.

## Background

In recent years, the new generation of analytical technology based on nanopores or nanochannels provides possibilities for low-cost and rapid biosensing and DNA sequencing. It is regarded as an emerging field which is expected to have major impact on bio-analysis and fundamental understanding of nanoscale interactions down to single-molecule level. Nowadays, more and more theoretical and experimental work aiming to understand and design nanopore-based devices have been done, which is at the forefront of life science, chemistry, material science, and (bio) physics. Among these studies, nanopore fabrication and nanopore-based nanofluidic device design are two key issues [1-4]. Generally speaking, there are two major types of nanopores which have been used for DNA sequencing and biosensing applications (e.g., using nanopores as analytical sensors for molecular or biomolecular analytes): protein nanopores [5-8] (such as the α-hemolysin pore) and artificial pores in solid-state films (such as silicon nitride nanopores [9-13], graphene nanopores [14-16], and silicon oxide nanopores [17,18]).

**Figure 1:**
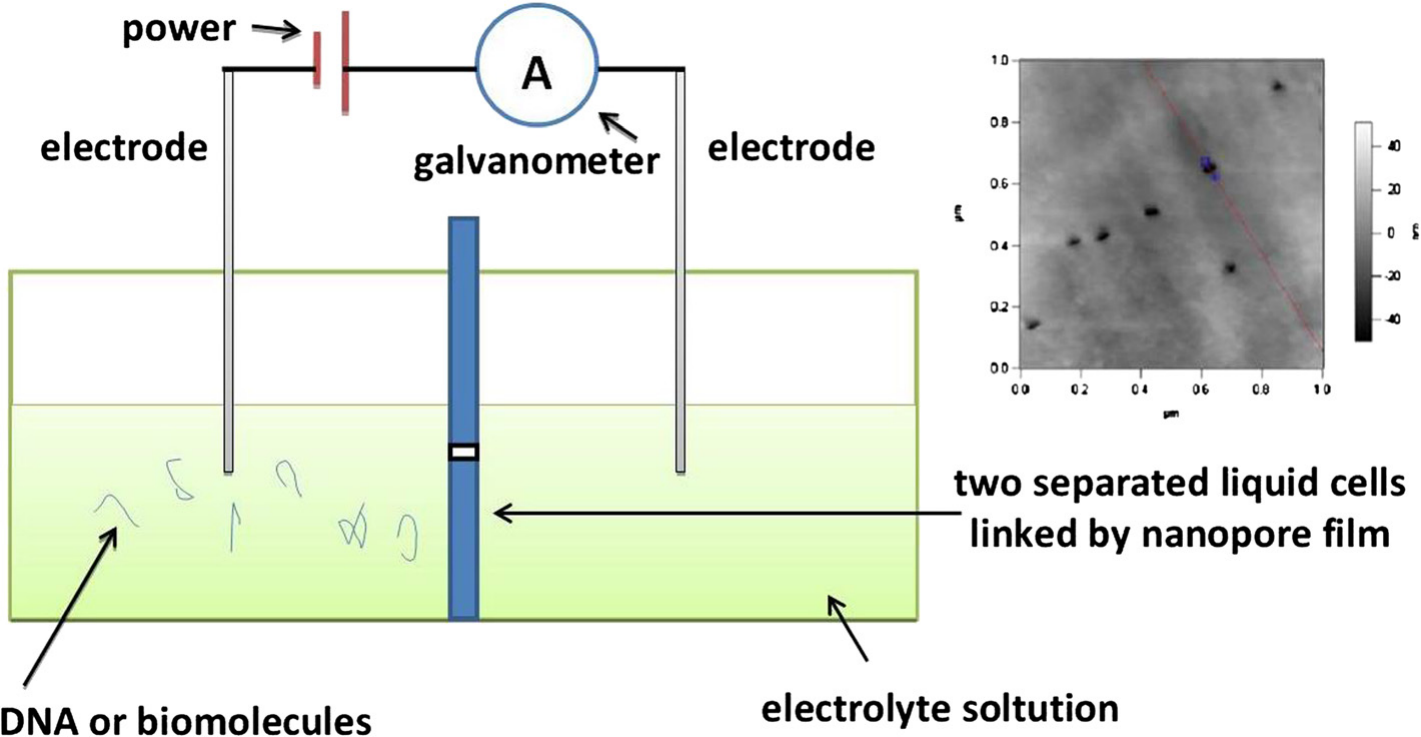
**The prototype nanofluidic device and AFM image of PC nanopore arrays.** The prototype nanofluidic device based on nanopores for single DNA sequencing or biomolecular sensing; and the AFM image of PC nanopore arrays is showed in the top right corner.

The scheme of nanofluidic analytical device (Figure 1) based on nanopores for biomolecular sensing can be simply depicted as following: two separated liquid cells with certain electrolyte are linked by nanopores; along the length direction of nanopore, certain voltage is applied, which results in background ionic current. Analytes in the electrolytic solution are electrophoretically driven through nanopores. When analytes pass through nanopore, they will cause changes in the background ionic currents. By the changes, the location and spatial structure of analytes in the nanopores can be determined. According to the existed research work, the molecular or macromolecular analytes possessing dimensions comparable to the size of nanopore are advantageous to get momentary ionic blockades with high signal-to-noise ratio. The concentration of the analytes in the liquid cell also can be distinguished from the frequency of these translocation events, and the structural information of the analytes can be encoded by analyzing the magnitude, duration, and shape of the current blockades. In addition, resistive-pulse sensing also can be used for the detection and characterization of ions and biopolymers [19-23].

Although much progress has been achieved in nanopore techniques, it is still difficult to sense nucleotides at single-base resolution in DNA. That is mainly because the thickness of nanopores (about several nanometers) can permit 10 to 15 nucleotides occupying them at one time. On the other hand, the momentary change in ionic currents is at only nano-ampere or pico-ampere level, and the duration of this change is at millisecond or so, which is hard to detect and analyzed. To improve the intensity of signals is an important issue in this area. Nanopore arrays, which can be regarded as the integration of multiple nanochannels in the same direction, can improve the intensity of signals in ionic current changes compared to single pore. Now, nanopore arrays are widely used in biomolecular separation, detection and analysis, although it seems difficult for DNA sequencing at present. In this work, the single molecule translocation properties through polycarbonate nanopore arrays are studied and discussed.

## Methods

### Experimental device and reagent

Polycarbonate membranes containing nanopore arrays (nanopore diameter 50 nm, nanopore distribution density 6 pores/μm^2^, thickness of polycarbonate membranes 6 to 11 μm) are purchased from the branch in China of Whatman, Inc. (Shanghai, China), and hydrophilic treatments are carried out before its usage. Goat antibody to human immunoglobulin G (IgG) is imported from America Basic Gene Associate Bioscience, Inc. through Nanjing Boquan Technology Co., Ltd. (Nanjing, China). KCl is commercially available, and it is of analytical grade. Ultra-pure water (resistivity 18.25 MΩ·cm) is used for the preparation of all solutions and rinsing. Keithley 2000 61/2-digital multimeter (Keithley Instruments Inc., Beijing, China) is used for ionic current recording. The applied voltage used in the experiments is varied 0.5 to 2V. AFM image in tapping mode is obtained from MFP-3D-SA atomic force microscope produced by Asylum Research (Santa Barbara, USA), and the scanning rate is 1.0 Hz.

A test device (Figure 1) integrated by two separated liquid cells linked by PC membrane containing nanopore arrays (sealed by PDMS) is used for measuring ionic currents. At room temperature, KCl solution is added to the feed cell and permeation cell, and IgG is dissolved in the reservoir. After that, the electric field is applied to the two sides of the membrane, and the trans-membrane ionic current can be measured by Keithley 2000 61/2-digital multimeter and recorded simultaneously by computer.

### Simulation model

**Figure 2:**
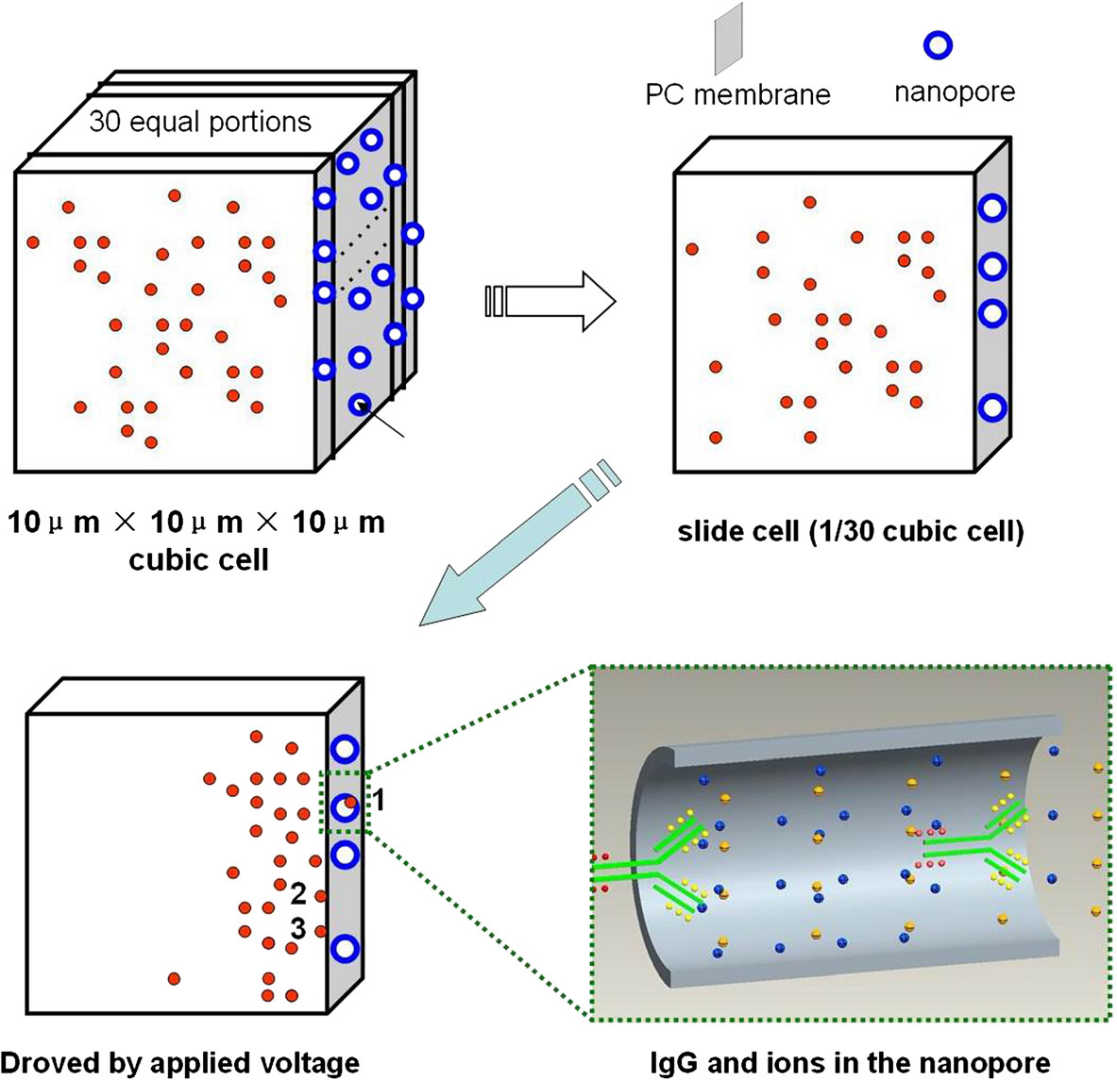
**The illustration of simulation cell.** The biomolecules are simplified as small balls in the solution; cubic cell with its side length of 10 μm close to the feed reservoir and divide the cubic cell equally into 30 slides along the *x* direction.

A simple model is suggested to depict IgG molecules passing through nanopore arrays. IgG molecules are simplified as small balls in the solution; the position, velocity, and acceleration of which in the solution are mainly determined by the electrostatic force between biomolecules, electrostatic force generated by the applied electric field, and van der Waltz force among the molecules (rather smaller). Select one cubic cell with its side length of 10 μm close to the feed reservoir, and divide the cubic cell equally into 30 slides along the *x* direction, as illustrated in Figure 2. The parameters for simulation are listed as Table 1. The program for the simulation is written in C++, and it is compiled and run on Borland C++ Builder (Micro Focus, Beijing, China).

**Table 1:** Parameters for simulation

**Items**	**Parameter setting**
Biomolecules	Relative molecular mass 140 kDa, surface charge density *σ* = 2.0 × 1,017/m^2^, concentration 10 ng/mL
Nanopore arrays in PC membrane	Pore diameter 50 nm, pore density 6 pores/μm^2^, membrane thickness 6 to 11 μm; its effective contact area contacting the solution is around 7 mm
Conditions	The applied electric field *E* = 0.1 V/nm, 0.1 M KCl solution

## Results and discussions

### The experimental approach

**Figure 3:**
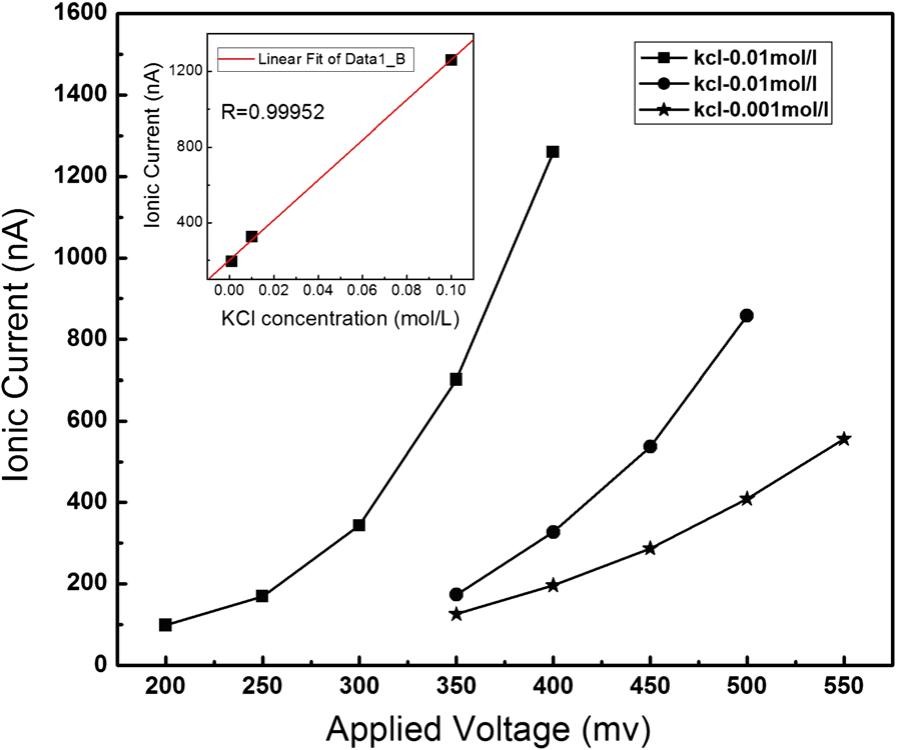
**The recorded ionic current increase with the applied voltage increasing.** The concentrations of the electrolyte solutions are 0.1, 0.01, and 0.001 mol/L, respectively, and the nanopore arrays with the diameter of 50 nm.

In our experiments, 0.001, 0.01, and 0.1 mol/L KCl aqueous solutions are employed as electrolytes for IgG detection. The pH value of the solution is controlled at 7.48 to guarantee the surface charge of IgG molecules being positive. When a certain voltage is applied to the two liquid cells through Pt electrodes, K^+^ and Cl^−^ are driven to pass through nanopores, which result in certain background ionic currents. As illustrated in Figure 3, the ionic current will increase with the increasing driven voltage if the concentration of KCl solution remains unchanged. It is obvious that bigger voltage corresponds to bigger electrostatic force, which will accelerate the movements of K^+^ and Cl^−^ and will lead to rather bigger ionic currents. On the other hand, if the driven voltage remains unchanged, the bigger density of ions in the solution will result in bigger ionic currents. For example, when the driven voltage is fixed at 400 mV, the ionic current is 1,260, 327, and 196 nA, corresponding to KCl concentrations of 0.1, 0.01, and 0.001 mol/L, respectively. From the inset picture in Figure 3, it can be found that the ionic currents rise linearly with the concentrations of electrolyte solution. These results indicate that the device based on nanopore arrays can be used for ionic current recordings.

**Figure 4:**
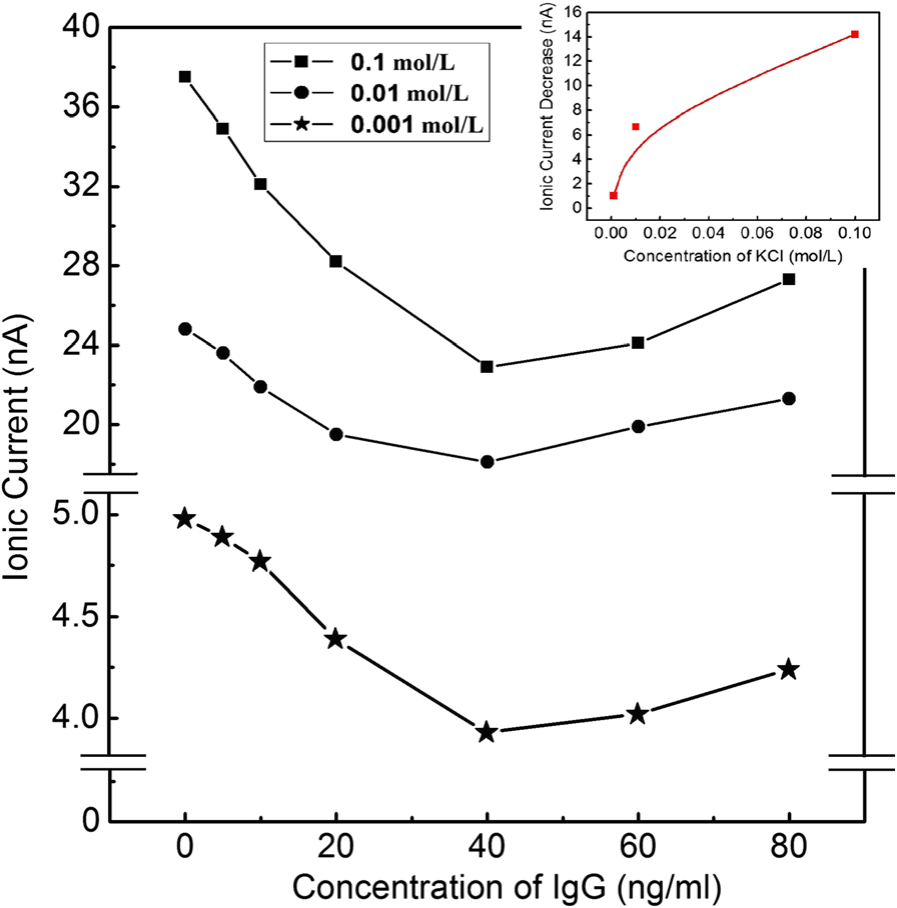
**Experimental results of the ionic current variation with IgG concentration in 0.1 mol/L KCl solution.** The applied voltage is 500 mV. The nanopore arrays possess the diameter of 50 nm.

**Figure 5:**
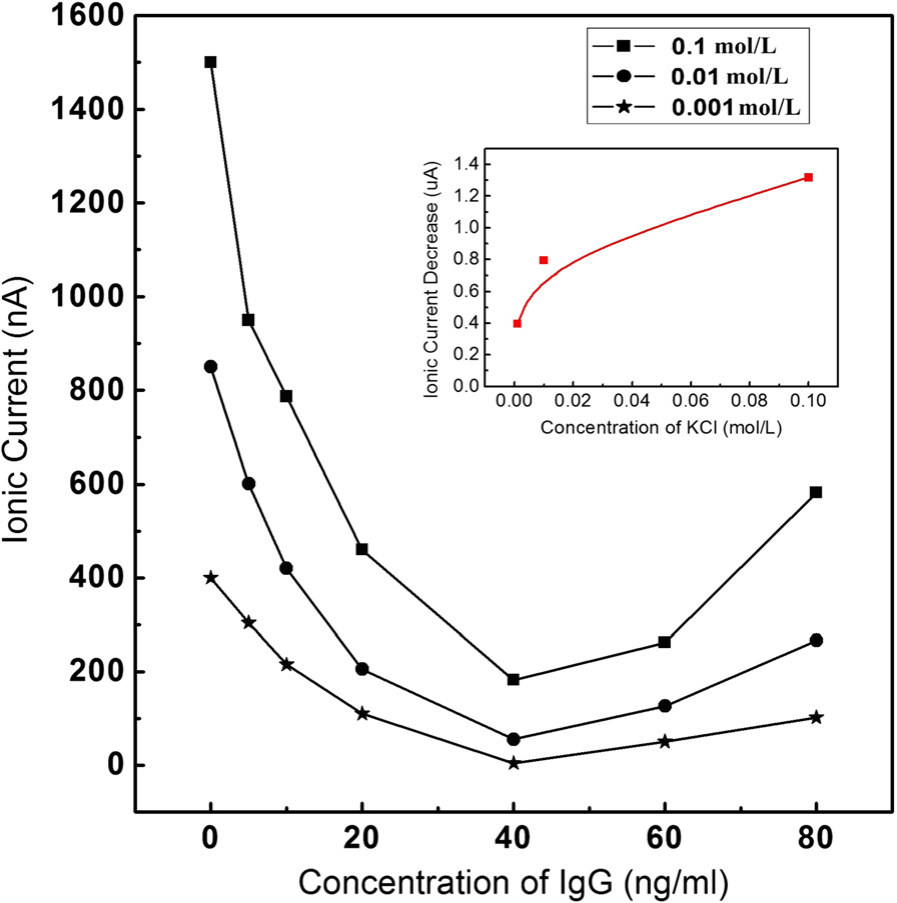
**Experimental results of the ionic current variation with IgG concentration in 0.1 mol/L KCl solution.** The applied voltage is 2 V. The nanopore array diameter is 50 nm.

When IgG molecules are added into the KCl solution, they are driven to pass through the nanopore arrays by the electrostatic force. The directional movements of IgG can change the original background ionic currents generated by the directional migrations of K^+^ and Cl^−^ and then result in the modulated ionic currents. Figures 4 and 5 show the ionic currents changing tendency with IgG concentrations increasing; the driven voltages are 500 mV and 2 V, respectively. In each picture, there are three curves from top to bottom, which represent the cases of KCl concentration at 0.1, 0.01, and 0.001 mol/L, respectively. From these results, it can be concluded that when the concentration of IgG is lower than 40 ng/mL, the ionic current will be decreased with the increase of the IgG concentration.

Generally, the change in the ionic current will be mainly affected by two factors: (1) physical place-holding effect. Once IgG molecules enter the nanopores, the volumes in the nanopores are partially occupied, which will prevent certain amounts of K^+^ and Cl^−^ from passing through PC membrane. It is so-called physical place-holding effect, and it will decrease the background ionic current. (2) Surface charge density of IgG molecule: as we know, the surface charge of IgG molecule will also contribute to the increase of total ionic current when it passes through the nanopore. The final current changes will be determined by the combined effects of the above two factors. When the concentration of electrolyte is quite higher, the density of anions and cations in the solution is also higher, and the lost number in anions and cations due to the physical place-holding effect is quite bigger. At the same time, the surface charge density of IgG molecules does not change if the pH of the solution remains at 7.48. In this condition, the decrease in ionic current generated by physical place-holding effect is bigger than the increase due to the contribution of IgG surface charge; so, there will be a decrease blockade in the background ionic current. When the concentration of electrolyte is quite lower, so that the decrease in current generated by physical place-holding effect is smaller than the increase in current due to the contribution of IgG surface charge, there will be an increase blockade in the background ionic current.

Based on the above analysis, the physical place-holding effect will be enhanced with the increasing concentration of IgG molecules in the solution within certain ranges; on the other hand, the volume of IgG molecule (IgG is one kind of molecule with “Y”-type structure and its size is about 20 nm) is much larger than K^+^ and Cl^−^, so the bulk charge density is much lower in the occupied nanopore arrays, which results in the decrease of ionic current. Of course, the modulated ionic current is affected not only by the physical place-holding effect but also by many other factors (such as electric double layer effect), so the decrease will nonlinearly change with the concentration of KCl. The differences between the background currents and the recorded currents at 40 ng/mL of IgG are plotted versus the concentration of KCl (insets of Figures 4 and 5), from which it can be found that the difference of current increase does ‘not’ linearly rise with the concentration of electrolyte.

According the above analysis and common sense, the current should continue to decrease along with the increasing concentration of IgG, but abnormal phenomenon appears when the concentration of IgG is higher than 40 ng/mL: the ionic currents do not decrease but increase with increasing IgG concentration. Undoubtedly, the physical place-holding effect also exists at these concentrations. The experimental results show that when IgG concentration is high enough, the translocation probability will not always increase with increasing IgG concentration. This is just like the following case: imagine a stadium with limited doors, the maximum allowed flux of people in unit time is *N*. When the number of people who need to enter the stadium is lower than *N*, the number of entering people will increase with the number of people who need to enter. If the number of people who need to enter the stadium in unit time is larger than *N*, the actual number of entering people will equal to or less than *N* (especially for disordered case). When IgG concentration is higher than a certain value (threshold value), the number of passing molecules will remain or be decreased. The physical place-holding effect is weakened, which will result in the ‘abnormal’ increase in the ionic current. The further explanation from the view of simulation is suggested in the following part.

### The simulation approach

The calculated results based on the suggested model are the outputs of the program after running 10,000 steps, which correspond to the number of IgG molecules passing through the nanopores in 10 ps. These obtained numbers in each step are discrete, but the numbers of passing IgG molecules in unit time can be regarded as the IgG moving velocity in the nanopores if the thickness of the nanopores is ignored. To simplify the calculation, we suppose that the nanopores move only in single row direction; the biomolecules passing through the nanopores can be investigated from a quasi two-dimensional perspective. In this slide cell, the acceleration of biomolecules is determined by total force, and then the velocity and position are determined. In one limited cell, the periodic boundary conditions are applied to guarantee the number of biomolecules in the cell being constant. The starting status is that IgG molecules are distributed homogeneously in the solution; as time goes on, the molecules in the solution become increasingly chaotic states, which are more close to the actual situation of the molecule movements and distributions. Therefore, only the last 5,000 steps are adopted and averaged of molecules in order to understand the change tendency of the number of molecules passing through the nanopores in unit time.

**Figure 6:**
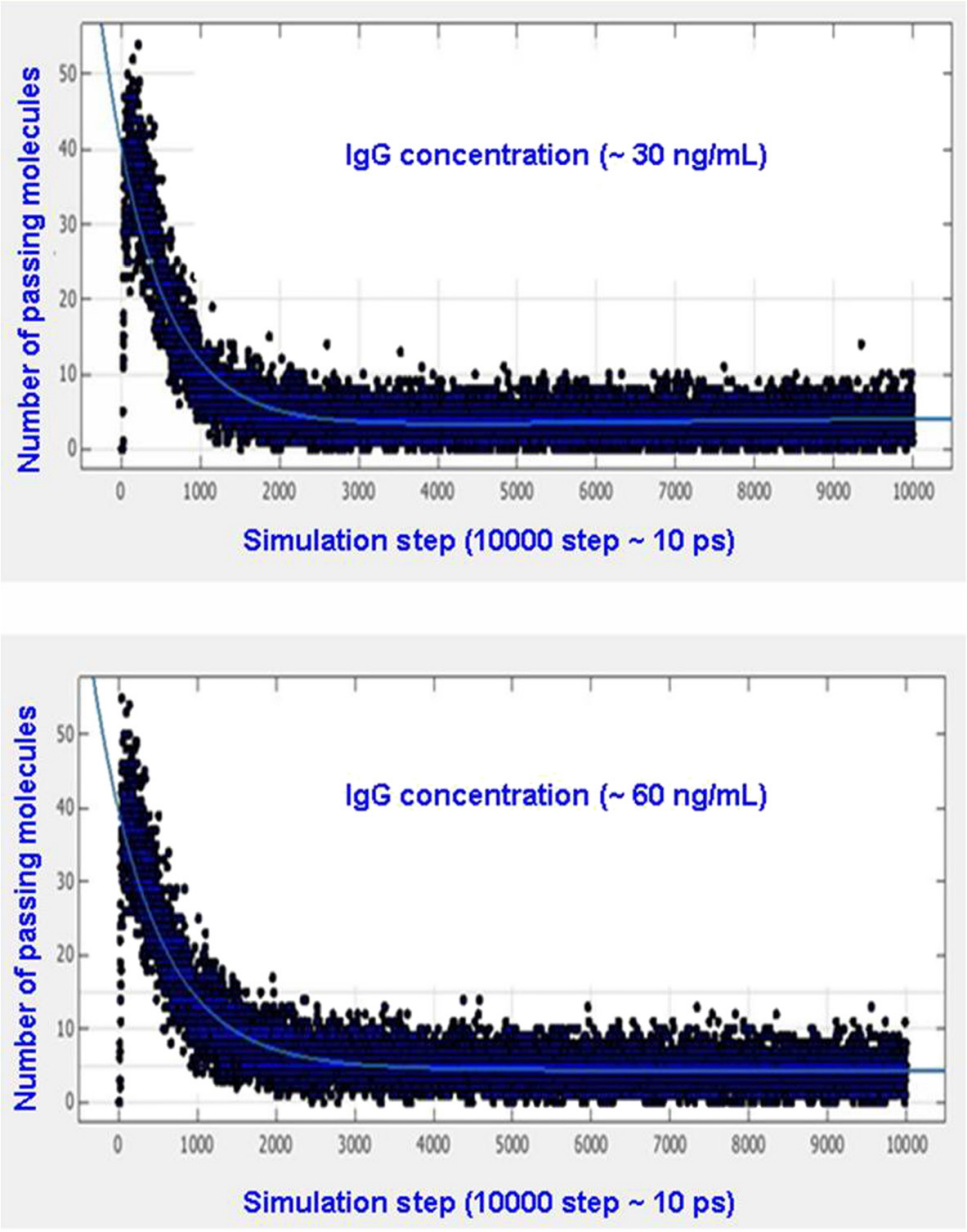
**Two cases of the calculated number of biomolecules passing through the nanopores.** IgG concentrations are about 30 and 60 ng/mL).

**Figure 7:**
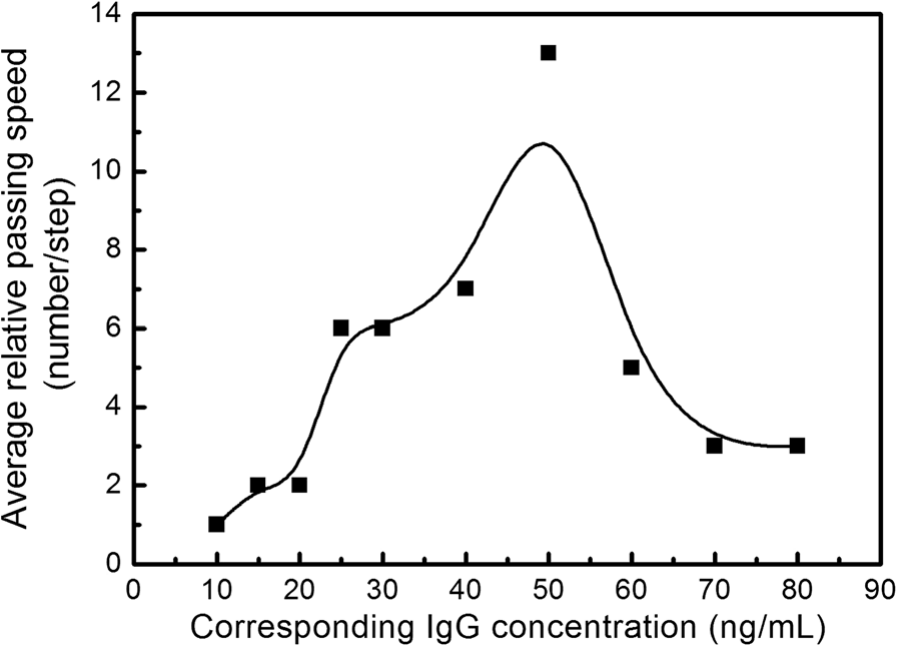
The calculated passing velocities of IgG molecules changing with IgG concentration.

Figure 6 shows the simulative results for IgG concentrations of 30 and 60 ng/mL. Solid black points stand for the number of IgG molecule passing the nanopores in one simulation step (10,000 step approximately 10 ps) and the blue line in the points is the average curve which corresponds to the average passing velocity of IgG. In this way, other velocities at different IgG concentrations can be obtained (the detailed results can be found in Additional file 1), and the calculated passing velocities of IgG molecules changing with IgG concentration can be plotted as showed in Figure 7. It can be found that with the increasing IgG concentration, the calculated passing velocity (the passing number in one simulative step) of biomolecules will not increase continuously but will increase at first, then will decrease and will finally stabilize. Considering the physical place-holding effect and the simulation results above, it can be predicted that with increasing IgG concentration, the ionic current will first decrease, then increase and finally stabilize. These conclusions provided support to our experimental results shown in Figures 4 and 5.

## Conclusions

In summary, the transporting properties of IgG molecules are investigated using nanopore arrays. The experimental results indicate that the ionic currents do not decrease continuously with increasing IgG concentration, as general consideration; the current decrease at first, then increase, and stabilize with the increasing concentration. The calculated passing velocity of IgG molecules based on a simplified model will first increase, then decrease, and finally stabilize with the increasing IgG concentration, which can provide support for our experimental results.

## Competing interests

The authors declare that they have no competing interests.

## Authors' contributions

LL carried out the experimental design and data analysis, and drafted the manuscript. BW, JS, and YY carried out the experimental work. YH, ZN, and YC participated in the theoretical studies. All authors read and approved the final manuscript.

## Supplementary Material

Additional file 1 Simulation model and results. Click here for file
