# Supplementary material for: Voltage-driven translocation behaviors of IgG molecule through nanopore arrays
Source: Nanoscale Res Lett. 2013 May 15;8(1):229. doi: 10.1186/1556-276X-8-229 (PMC3664219; doi:10.1186/1556-276X-8-229)
Supplement: Additional file 1 — Simulation model and results. [file 1556-276X-8-229-S1.doc]

**Supplementary Data**

**Simulation Model**


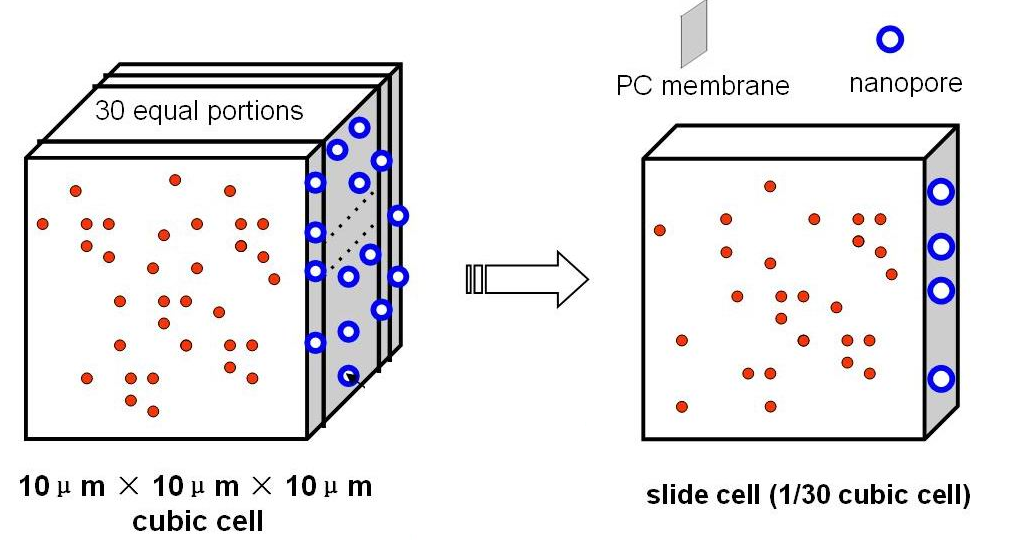


Figure 1

As showed in Fig. 1, IgG molecules are simplified as small balls, whose position, velocity and acceleration in the solution are mainly determined by the electrostatic force between biomolecules, electrostatic force generated by the applied electric field, and van der Waltz force among molecules (rather smaller). Select one cubic cell with its side length of 10μm close to the feed reservoir, and divide the cubic cell equally into 30 slides along x-direction. The parameters for our simulation are listed as followings:

| Items | Parameter setting |
| --- | --- |
| Biomolecules | Relative molecular mass:140kDa; Surface charge density: σ=2.0×10^17^/m^2^; concentration 10ng/mL |
| Nanopores arrays in PC membrane | pore diameter: 50nm; pore density: 6 pores/μm^2^; membrane thickness 6-11μm. Its effective contact area contact to the solution is a round with diameter of 7 mm |
| conditions | The applied electric field E=0.1V/nm, 0.1 M KCl solution |

**Simulation Results**

**Fig. 2** to **Fig. 10** show the computational results based on the above model for nine kinds of cases, corresponding to the IgG concentration of 10 ng/mL, 15 ng/mL, 20 ng/mL, 25 ng/mL, 30 ng/mL, 40 ng/mL, 50 ng/mL and 60 ng/mL and 80 ng/mL respectively. In the following figures, solid black points stand for the number of IgG molecule passing nanopres in one simulation step (10000 step ~10 ps), and the blue line in the points is the average curve, which corresponds to the average passing velocity of IgG.


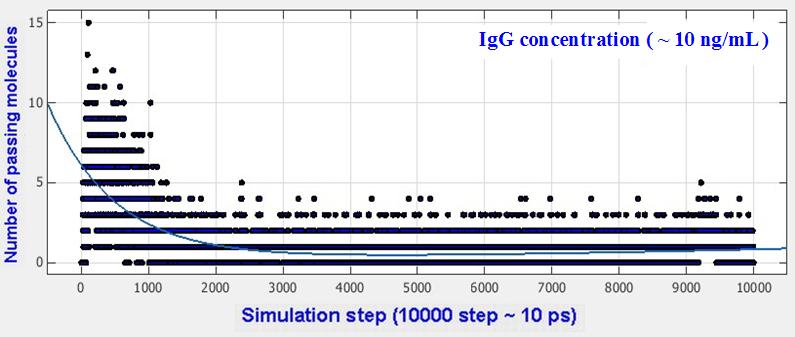


Figure 2


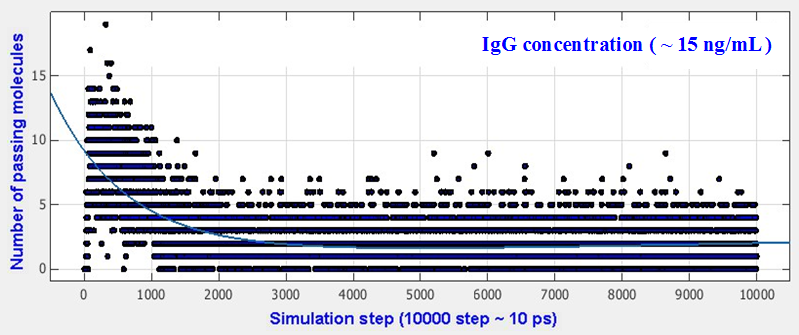


Figure 3


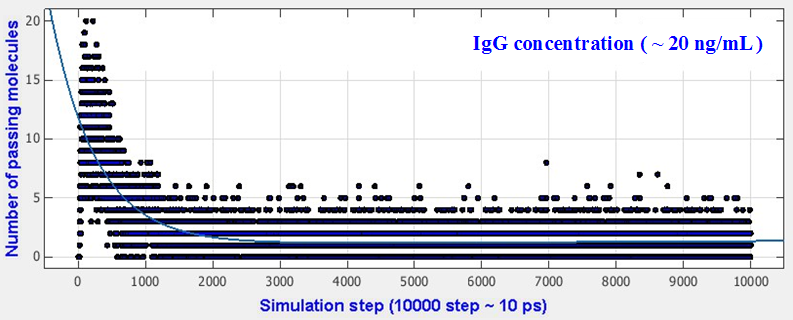


Figure 4


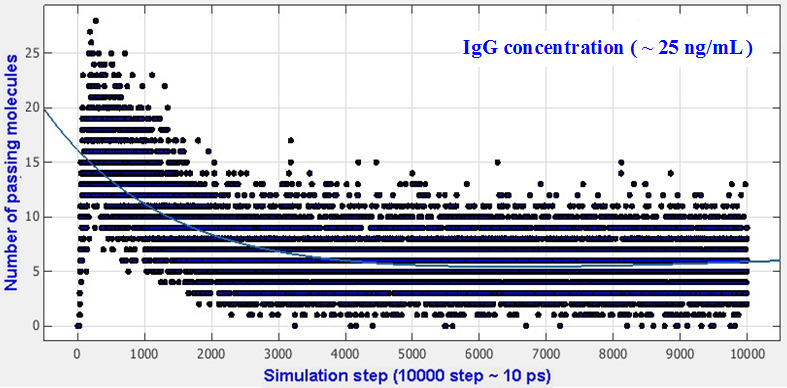


Figure 5


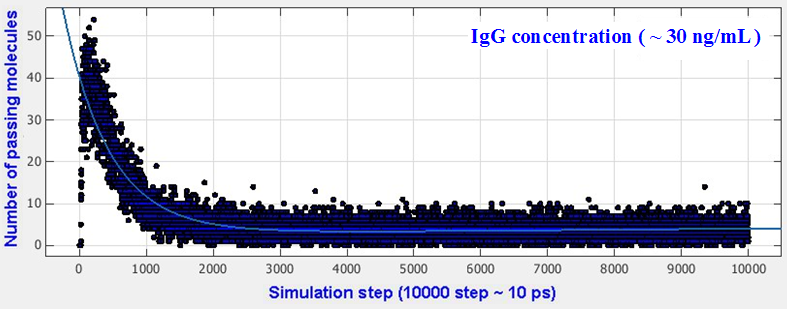


Figure 6


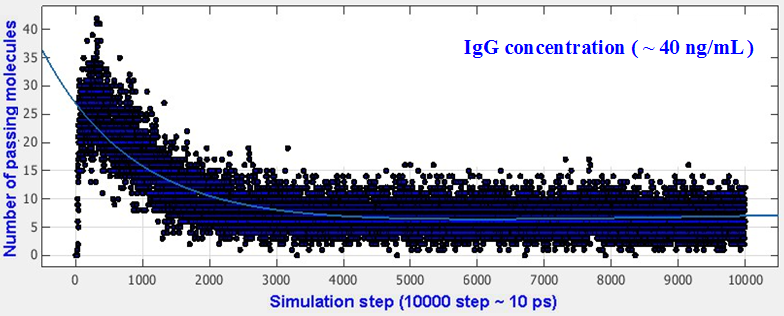


Figure 7


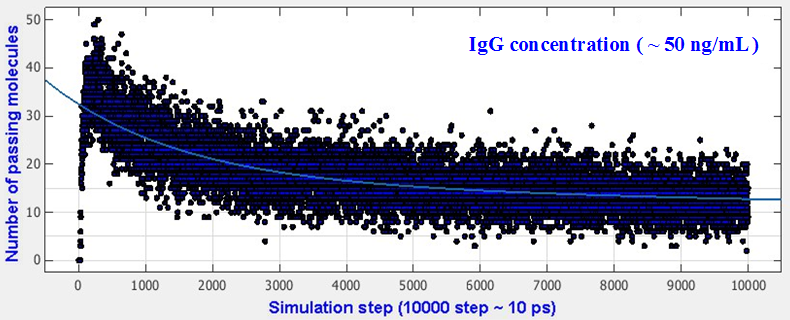


Figure 8


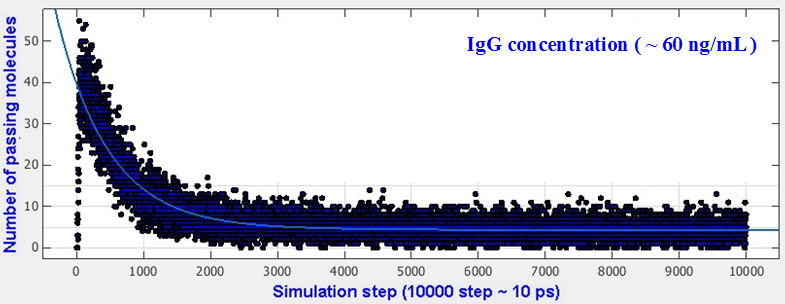


Figure 9


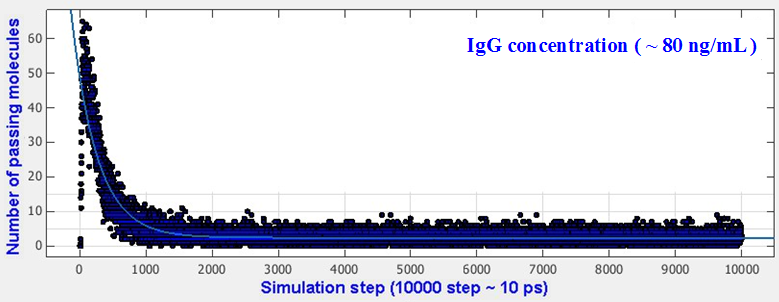


Figure 10

Based on the above computational data, the calculated passing velocities of IgG molecules changing with IgG concentration can be plotted as Fig.11


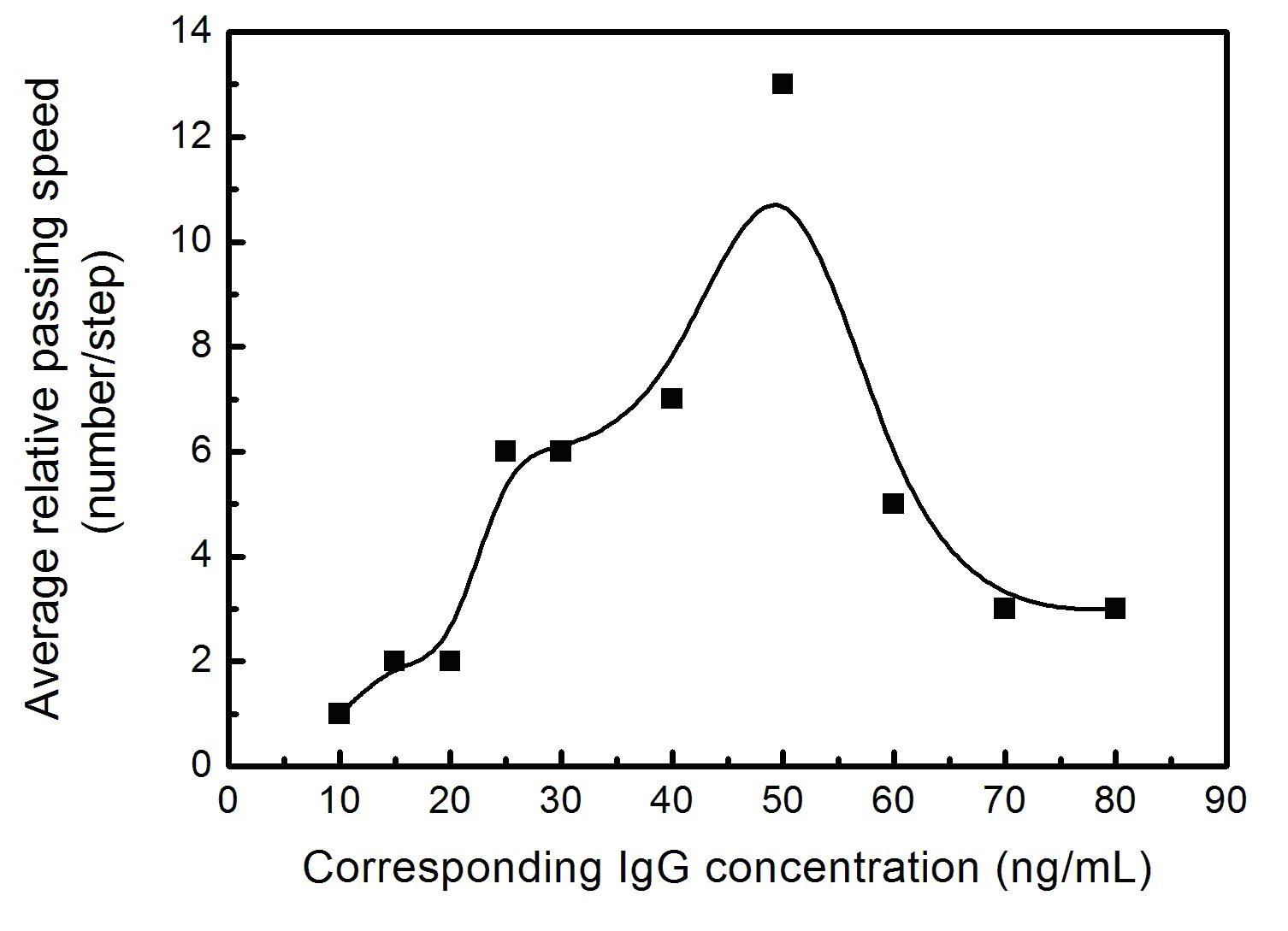


Figure 11
